# Atezolizumab-Induced Autoimmune Diabetes in a Patient With Metastatic Lung Cancer

**DOI:** 10.3389/fendo.2019.00352

**Published:** 2019-06-11

**Authors:** Jin Sothornwit, Anakapong Phunmanee, Chatlert Pongchaiyakul

**Affiliations:** ^1^Division of Endocrinology and Metabolism, Department of Internal Medicine, Faculty of Medicine, Khon Kaen University, Khon Kaen, Thailand; ^2^Division of Critical Care, Department of Internal Medicine, Faculty of Medicine, Khon Kaen University, Khon Kaen, Thailand

**Keywords:** atezolizumab, autoimmunity, checkpoint inhibitor, diabetes mellitus, immunotherapy

## Abstract

**Context:** Immune checkpoint inhibitors (ICIs), now FDA-approved, are increasingly used as an effective treatment of various cancers. Autoimmune diabetes is a rare but life-threatening endocrine adverse event, which has been reported in patients treated with anti-programmed-cell death-1 (anti-PD-1) and anti-programmed-cell death-1 ligand (anti-PD-L1) therapies.

**Case description:** We report a 52-year-old woman with advanced-stage non-small cell lung cancer who presented with diabetic ketoacidosis (DKA) at 24 weeks after atezolizumab initiation. She initially received oral antidiabetic medication from primary care hospital and experienced recurrent DKA 3 days later. Her plasma glucose on the day that she had recurrent DKA was 332 mg/dL (18.4 mmol/L), A1c was 7.9% (63 mmol/mol), fasting C-peptide was <0.03 nmol/L (0.1 ng/ml), fasting insulin level was <1 μIU/ml, anti-glutamic acid decarboxylase 65 (GADA) was 7.2 U/ml (normal, >5 U/ml), and human leukocyte antigen (HLA) class II typing was DR3-DQ2/DR14-DQ5. A diagnosis of autoimmune diabetes was made. After treatment for DKA, she recovered and received basal-bolus insulin treatment. Atezolizumab had been discontinued after the fifth cycle, prior to the development of DKA, due to progression of lung cancer.

**Conclusion:** To date, there has been neither an effective way to detect if a patient is at high risk for autoimmune diabetes nor to prevent the complications associated with it. Regular glucose monitoring is the best method of early diabetes detection. In patients with new onset diabetes following treatment with ICIs, C-peptide levels and GADA should be screened, and insulin therapy should be prescribed to prevent hyperglycemic emergency while waiting for definite diagnosis.

## Introduction

Immune checkpoint inhibitors (ICIs) are now approved for the treatment of various cancers. These medications improve survival outcomes and provide a potentially curative treatment option by removing inhibitory signals of T-cell activation, which enables tumor-reactive T cells to overcome regulatory mechanisms and produce an effective antitumor response. However, immunologic tolerance can be altered, which results in immune-related adverse events including gastrointestinal, dermatologic, pulmonary, and endocrine adverse effects (1). Among other immune-related adverse events (irAEs), thyroid disorders and hypophysitis are common endocrinopathies that result from immunotherapy. But rarer endocrinopathies (i.e., primary adrenal insufficiency and autoimmune diabetes mellitus), have also been reported, particularly in patients receiving anti-PD-1 or anti-PD-L1 (2).

## Methods

This study was carried out in accordance with the recommendations of Center for Ethics in Human Research, Khon Kaen University with written informed consent from subject. Subject gave written informed consent in accordance with the Declaration of Helsinki for the publication of this case report and any potentially-identifying images/information.

## Case Presentation

**Figure 1:**
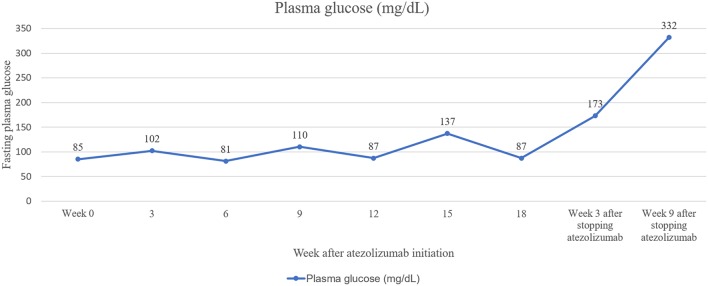
Plasma glucose during treatment with atezolizumab.

A 52-year-old Thai female was diagnosed with stage 4 lung adenocarcinoma with adrenal metastases, T4N3M1b. Epidermal growth factor receptor (EGFR) and anaplastic lymphoma kinase (ALK) mutation analyses were negative. Programmed death-ligand 1 (PD-L1) expression on tumor cells was more than 1%. She received 1,200 mg of atezolizumab every 3 weeks for 5 cycles. She achieved a partial response by 12 weeks after therapy, then the medication was discontinued after 18 weeks of treatment due to disease progression. She had no other underlying diseases and no family history of diabetes and other autoimmune disease. Her fasting plasma glucose was 85 mg/dL (4.7 mmol/L) before atezolizumab initiation (plasma glucose levels during therapy are shown in Figure 1). She presented with diabetic ketoacidosis (DKA) at 24 weeks after the first dose and 9 weeks after cessation of atezolizumab. She was first diagnosed with diabetes with an A1c of 7.9% (63 mmol/mol) and was discharged from primary care hospital with glipizide. Three days after discharge, she was admitted to our hospital with severe DKA. Her initial serum glucose was 332 mg/dL (18.4 mmol/L) and A1c was 7.9%. She had wide gap metabolic acidosis with serum bicarbonate of 9.9 mEq/L, anion gap of 24.1, and the arterial pH of 6.9. Her serum β-hydroxybutyrate was 5.91 mmol/L, and lactate was 1.06 mmol/L. There was no infection, thromboembolic event, or medication causing hyperglycemia. Atezolizumab-induced autoimmune diabetes was suspected. At 7 weeks after DKA, fasting C-peptide was <0.03 nmol/L (0.1 ng/ml) and fasting insulin level was <1 μIU/ml while plasma glucose was 380 mg/dL (21.1 mmol/L). Anti-glutamic acid decarboxylase 65 (GADA) and anti-tyrosine phosphatase-like insulinoma antigen 2 (anti-IA2), measured by enzyme-linked immunosorbent assay (ELISA) method, were positive (7.2 U/ml; >5 U/ml) and negative (<7.5 U/ml), respectively. We did not test for anti-Zinc transporter isoform 8 (ZnT8) and anti-insulin (IAA) since the tests were unavailable in our country. The results of HLA class II typing by sequence-specific oligonucleotide primed PCR were DRB1^*^03, DRB1^*^14, DQB1^*^02, and DQB1^*^05 (DR3-DQ2/DR14-DQ5). She was being treated with basal-bolus insulin therapy, consisted of once-daily basal insulin glargine (Lantus®) plus thrice-daily prandial insulin aspart (Novorapid®), with a total daily insulin dose of 0.5 units per kilogram per day. Her thyroid function tests, both before and after receiving atezolizumab, and the levels of other anterior pituitary hormones after receiving atezolizumab were normal, as shown in Table 1. She had other adverse immune-associated reactions during the first cycle of therapy, including neuralgia grade 1 and transaminitis grade1, which resolved spontaneously after 3 weeks. Her lung cancer was then treated with paclitaxel and carboplatin leading to partial remission.

**Table 1:** Levels of anterior pituitary hormones and diabetes-relevant laboratory results in this patient before and after initiation of atezolizumab.

**Laboratory results**	**Before atezolizumab initiation**	**31 weeks after atezolizumab initiation**
**DIABETES-RELEVANT LABORATORY RESULTS**		
FBS (mg/dL)	85	332
A1c (%)	No data	7.9
Insulin (μIU/ml)	No data	< 1
C-peptide (nmol/L)	No data	< 0.03
GADA (positive; > 5 U/ml)	No data	7.2
IA2 (positive; >7.5 U/ml)	No data	< 7.5
HLA class II typing		DR3-DQ2/DR14-DQ5
**ANTERIOR PITUITARY HORMONES**		
TSH (0.20-4.20 mIU/L)	1.45	0.936
FT4 (0.78-2.11 ng/dL)	1.22	1.05
FT3 (2.30-6.90 pg.ml)	2.87	2.18
Estradiol (women follicular phase 12.4-233 pg/ml, patient has had regular menstruation)	No data	98.63
FSH (mIU/ml)		16.00
LH (mIU/ml)		8.20
Prolactin (ng/ml)	No data	11.40
8 AM cortisol (ng/dL)	No data	16.00
ACTH (pg/ml)		21.00

## Discussion

Atezolizumab, a humanized monoclonal antibody to PD-L1, is one of the ICIs. This medication has received FDA approval for the treatment of patients with metastatic non-small cell lung cancer (NSCLC), locally advanced or metastatic urothelial carcinoma, and triple-negative breast cancer who experience disease progression after platinum-based chemotherapy. The recommended dose is 1,200 mg administered as an intravenous infusion every 3 weeks until disease progression or unacceptable toxicity (3).

Immune checkpoints are inhibitory signaling pathways in immune cells that maintain the peripheral tolerance of self-antigens. These pathways are regulated by receptor-ligand pairings (4). Programmed death receptor-1 (PD-1) is one of the inhibitory receptors that bind to programmed death-ligand 1 (PD-L1) (5). Tumor cells express immune checkpoint molecules and inhibit antitumor immune response. Moreover, ICIs enhance the activation of cytotoxic T-cells leading not only to antitumor immunity but also to autoimmunity. PD-L1 is expressed on pancreatic islet cells, providing a protective effect against β-cell autoimmunity. Therefore, this is one of the potential mechanisms that autoimmune diabetes mellitus may develop after administering anti-PD-1 or anti-PD-L1 (6). The association of new onset autoimmune diabetes following anti-PD-1 and anti-PD-L1 is supported by studies that have shown decreased PD1 expression of peripheral CD4+ T-cells in patients with type 1 diabetes (7) and selective β-cell destruction in non-obese diabetic (NOD) mouse models (8).

The onset of diabetes has been shown to range from 1 week to 228 weeks, with a median onset of 20 weeks after drug initiation (6, 9, 10). Our case had the diabetes onset at 24 weeks, which was close to the median onset. The loss of β-cell is acute, while other islet cells may be preserved, since random glucagon levels in patients with ICI-induced diabetes have been shown to be within the normal range (10). Although new onset autoimmune diabetes mellitus associated with ICI treatment is extremely rare, it often presents as DKA, a medical emergency requiring immediate treatment. Therefore, assessment of fasting plasma glucose every 3 months (or immediately in the case of onset of clinical signs), and ketone assessment if plasma glucose is higher than 250 mg/dL are recommended (9). First-line GADA screening should be performed in all cases. If these antibodies are absent, tests for anti-IA2, IAA, and ZnT8 antibodies may be conducted. However, lack of typical autoantibodies should not prevent the diagnosis of autoimmune diabetes in patients treated with ICIs. In our case, the patients' plasma glucose levels exhibited an upward trend. Early assessment and treatment with insulin in such cases may prevent hyperglycemic emergency. In addition to glucose measure, monitoring for C-peptide loss along with continuous glucose monitoring (CGM) may aid in early recognition of ICI-induced diabetes.

The risk factors for autoimmune diabetes are unknown. Presence of other autoimmune diseases or immune-related adverse events is not a risk factor for developing autoimmune diabetes (9). The association between HLA alleles and ICI-induced diabetes is controversial. Specific variation in HLA-I and HLA-II molecules, which present antigens to T-cells and initiate immune response, have been shown to be associated with increased risk of the development of type 1 diabetes, especially the HLA class II genes DR and DQ. The DR-DQ types that carry the highest risk for type 1 diabetes mellitus are HLA DR3-DQ2 and HLA DR4-DQ8 (11, 12). Stamatouli et al. found a predominance of HLA DR4 in patients with ICI-induced diabetes when compared with both the background population and type 1 diabetes patients. The frequencies of HLA-DR3 and HLA DQ8 (DQB1^*^0302), which were in linkage disequilibrium with HLA-DR4, also increased in immune checkpoint inhibitor-induced diabetes but were similar to those of type 1 diabetes patients (10). In our patient, HLA class II typing was DR3-DQ2, which carries with it a high risk for autoimmune diabetes, supporting the genetic risk hypothesis. But Lowe et al. found that some patients had HLA with a protective haplotype for developing type 1 diabetes (13), so additional investigation is needed. Although screening with HLA typing before ICI initiation is currently not recommended, HLA typing and pre-treatment autoantibodies screening may contribute in the long run to understanding of the range of the problem and connections between autoimmunity and cancer. Moreover, pre-treatment patients with potential susceptibility to autoimmune diabetes would raise awareness of the physicians administering ICI.

Like that of autoimmune diabetes, the treatment of ICI-induced diabetes consists of multiple-dose insulin injection. Immunosuppression with corticosteroids may not be useful since up to 80–95% of the pancreatic β-cell mass will have been permanently destroyed (4). Therefore, it is accepted that ICIs can be continued except in cases of drug toxicity or disease progression (4). No cases of remission of diabetes after cessation of ICIs have been reported (9). However, glucose should be monitored periodically in patients who have not developed autoimmune diabetes, since the autoimmune process may continue despite ICI termination (9). Further research is necessary to better understand the prognosis of cancer in patients with immunotherapy-induced autoimmune diabetes (9).

## Conclusion

Immune checkpoint inhibitors, especially anti-PD-1 and anti-PD-L1 and probably cytotoxic T lymphocyte antigen 4 (CTLA-4) inhibitors, do cause autoimmune diabetes. To date, there has been neither an effective way to detect if a patient is at high risk for autoimmune diabetes nor to prevent the complications associated with it. Regular glucose monitoring is the best method of early diabetes detection. In patients with new onset diabetes following treatment with ICIs, C-peptide levels and insulin autoantibodies should be screened, and insulin therapy should be prescribed to prevent hyperglycemic emergency while waiting for definite diagnosis.

## Ethics Statement

This study was carried out in accordance with the recommendations of Center for Ethics in Human Research, Khon Kaen University with written informed consent from subject. Subject gave written informed consent in accordance with the Declaration of Helsinki. The protocol was approved by Center for Ethics in Human Research, Khon Kaen University.

## Author Contributions

All authors listed have made a substantial, direct and intellectual contribution to the work, and approved it for publication.

### Conflict of Interest Statement

The authors declare that the research was conducted in the absence of any commercial or financial relationships that could be construed as a potential conflict of interest.
